# Metals from spacecraft reentry in stratospheric aerosol particles

**DOI:** 10.1073/pnas.2313374120

**Published:** 2023-10-16

**Authors:** Daniel M. Murphy, Maya Abou-Ghanem, Daniel J. Cziczo, Karl D. Froyd, Justin Jacquot, Michael J. Lawler, Christopher Maloney, John M. C. Plane, Martin N. Ross, Gregory P. Schill, Xiaoli Shen

**Affiliations:** ^a^Chemical Sciences Laboratory, National Oceanic and Atmospheric Administration, Boulder, CO 80305; ^b^Department of Earth, Atmospheric, and Planetary Sciences, Purdue University, West Lafayette, IN 47907; ^c^Cooperative Institute for Research in Environmental Sciences, University of Colorado, Boulder, CO 80309; ^d^School of Chemistry, University of Leeds, Leeds LS29JT, United Kingdom; ^e^The Aerospace Corporation, El Segundo, CA 90245

**Keywords:** stratosphere, aerosol, spacecraft, reentry, meteors

## Abstract

Large increases in the number of low earth orbit satellites are projected in the coming decades [L. Schulz, K.-H. Glassmeier, *Adv. Space Res.* **67**, 1002–1025 (2021)] with perhaps 50,000 additional satellites in orbit by 2030 [GAO, Large constellations of satellites: Mitigating environmental and other effects (2022)]. When spent rocket bodies and defunct satellites reenter the atmosphere, they produce metal vapors that condense into aerosol particles that descend into the stratosphere. So far, models of spacecraft reentry have focused on understanding the hazard presented by objects that survive to the surface rather than on the fate of the metals that vaporize. Here, we show that metals that vaporized during spacecraft reentries can be clearly measured in stratospheric sulfuric acid particles. Over 20 elements from reentry were detected and were present in ratios consistent with alloys used in spacecraft. The mass of lithium, aluminum, copper, and lead from the reentry of spacecraft was found to exceed the cosmic dust influx of those metals. About 10% of stratospheric sulfuric acid particles larger than 120 nm in diameter contain aluminum and other elements from spacecraft reentry. Planned increases in the number of low earth orbit satellites within the next few decades could cause up to half of stratospheric sulfuric acid particles to contain metals from reentry. The influence of this level of metallic content on the properties of stratospheric aerosol is unknown.

## Significance

Measurements show that about 10% of the aerosol particles in the stratosphere contain aluminum and other metals that originated from the “burn-up” of satellites and rocket stages during reentry. Although direct health or environmental impacts at ground level are unlikely, these measurements have broad implications for the stratosphere and higher altitudes. With many more launches planned in the coming decades, metals from spacecraft reentry could induce changes in the stratospheric aerosol layer.

Aerosol particles that are formed in the stratosphere are composed primarily of sulfuric acid derived from the oxidation of carbonyl sulfide and, after volcanic eruptions, sulfur dioxide (1). About half of the 120 to 600 nm diameter sulfuric acid particles contain small amounts, typically a few weight percent, of metals and silicon derived from meteoric ablation at altitudes above 75 km (2–5). If the sulfuric acid and associated water were removed, the remaining metals and silicon would be less than 100 nm in diameter. Other than the metals, the sulfuric acid in this type of stratospheric aerosol is typically extremely pure with minimal organic content. The sulfuric acid particles described here are distinct from both larger solid particles (6) that may represent the remnants of incompletely ablated meteoroids (7, 8) and from tropospheric particles transported into the stratosphere (9).

Air descends in the stratosphere near the North and South poles in winter for each hemisphere, making the high-latitude winter stratosphere a good location to see the influence of processes at yet higher altitudes. Flights of the NASA WB-57 from Fairbanks, Alaska, during the Stratospheric Aerosol processes, Budget and Radiative Effects (SABRE) mission in February and March 2023 sampled the high-latitude stratosphere at pressure altitudes up to 19 km. The high-altitude sampling along with descending air in that part of the stratosphere effectively excludes particles from lower altitudes. The Particle Analysis by Laser Mass Spectrometer (PALMS) instrument on the WB-57 obtained mass spectra of over 500,000 single aerosol particles during SABRE. Results from earlier missions are also included for comparison.

## Results

**Fig. 1.**
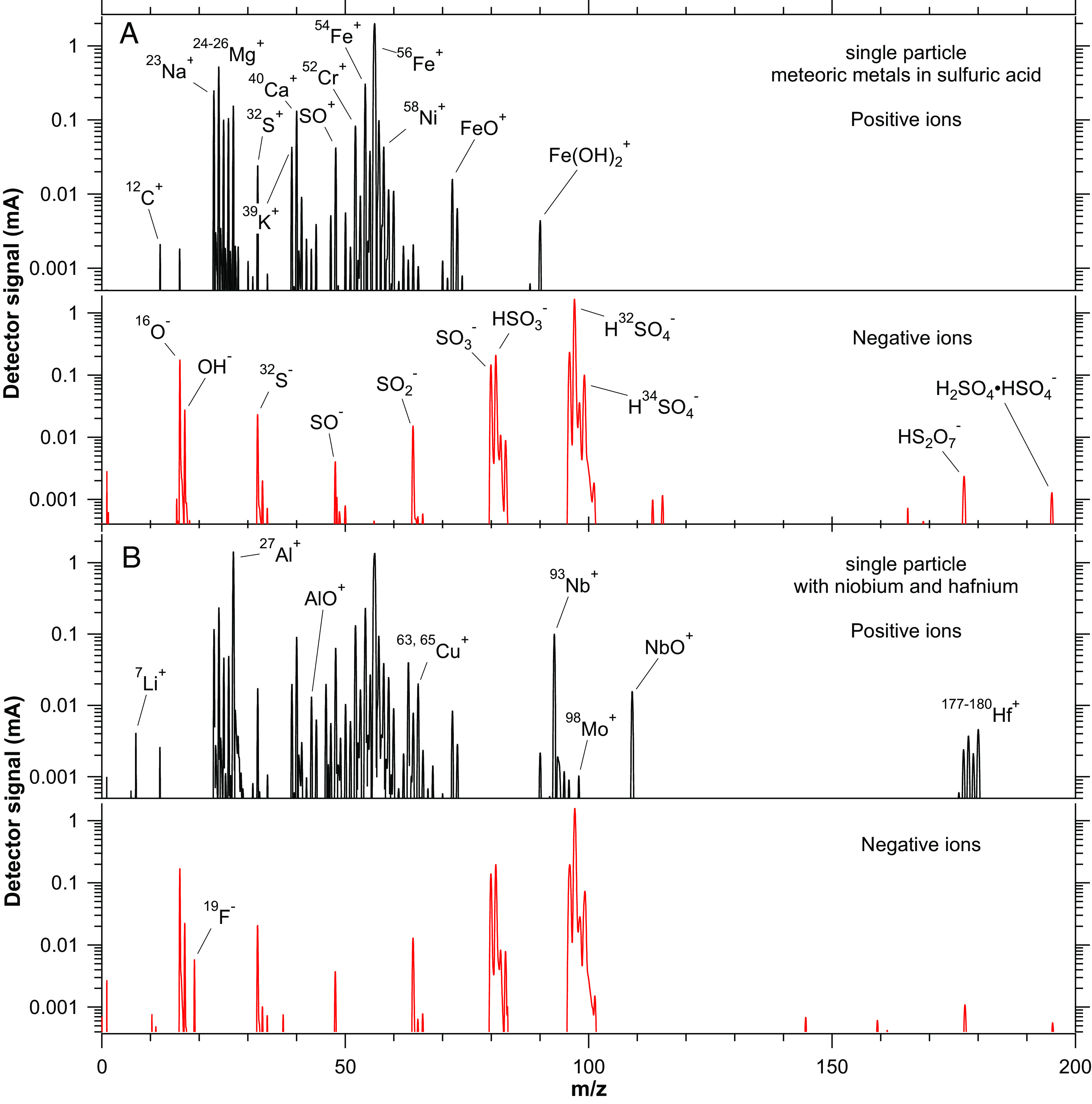
Examples of mass spectra of single particles in the stratosphere. These spectra were chosen to be representative of a typical sulfuric acid particle with meteoric metals (*A*) and a particle with both meteoric metals and metals from spacecraft reentry including niobium and hafnium (*B*). In (*B*), only peaks that are significantly enhanced are tagged.

Metals including Na, Mg, Cr, Fe, and Ni are present in many stratospheric particles in extremely consistent ratios and provide a clear signature of meteoric content. The metal atoms that are produced by meteoric ablation become oxidized below 85 km, and the resulting compounds condense into nm-sized meteoric smoke particles. The mass of the meteoric material in each stratospheric sulfuric acid particle is such that many meteoric smoke particles must have coagulated in order to form the material within that particle (3). More than one ablated meteoroid contributes to each stratospheric particle: There is, for example, no evidence of a subpopulation of sulfuric acid particles that formed on smoke particles from just iron-nickel meteoroids. Fig. 1*A* shows a single-particle mass spectrum of a typical sulfuric acid particle containing meteoric metals.

**Fig. 2.**
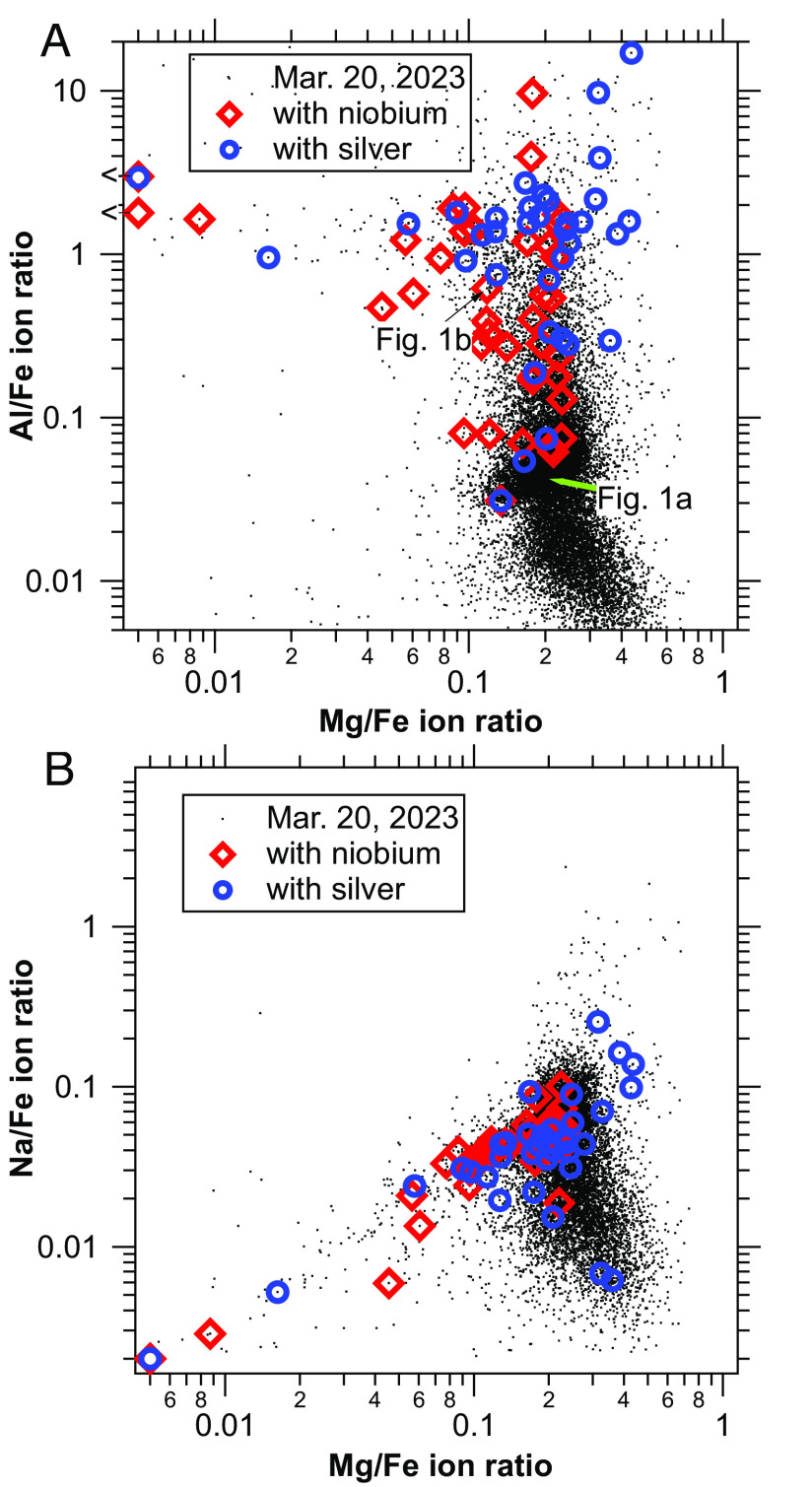
The magnesium, aluminum, and sodium ratios to iron peaks in sulfuric acid particles which contain iron. Each scatter plot uses data from only one flight (n ≈ 19,000) to avoid excessive overlap of points. (*A*) Al/Fe versus Mg/Fe. spacecraft. (*B*) Na/Fe versus Mg/Fe. Aluminum is a major component of spacecraft whereas sodium is not. Spectra with Nb or Ag are highlighted, showing that particles with nonmeteor elements consistently have more Al as well.

Fig. 2 shows the relationships between the sodium, aluminum, magnesium, and iron ion signals in stratospheric sulfuric acid particles. There is a dense cluster of points at an Mg/Fe ion ratio of about 0.2 and an Al/Fe ratio of about 0.04. Applying laboratory calibrations (*Materials and Methods*) to the ion ratios at the clusters in Fig. 2*A* yields meteoric mass ratios of 0.21 ± 0.1 for Mg/Fe and 0.015 ± 0.007 for Al/Fe. These may be compared to mass ratios of 0.35 and 0.018 from a semiempirical model of meteoroid ablation (8) and 0.27 and 0.009 from metal ions measured in the ionosphere above 90 km (10). Fig. 2*B* yields a meteoric mass ratio of 0.07 ± 0.035 for Na/Fe compared to 0.096 for the model of meteoroid ablation (sodium ion chemistry complicates a comparison to the ionosphere).

The tight clustering of the main groups of points in both panels of Fig. 2 suggests that the laser ionization sampled the entire particle. Points at lower Al/Fe or lower Na/Fe ratios are also from meteors and probably represent incomplete vaporization and ionization in PALMS (5). Besides the lower Al/Fe and Na/Fe ratios, these particles are larger than average and have lower-than-average total signal levels. If such particles are incompletely vaporized, then any inclusions or radial concentration gradients in the particles would change the magnitude and consistency of the ion ratios. However, it is difficult to exclude the possibility of a second population of meteoric particles with a different atmospheric history than the main cluster of points.

There are several lines of evidence that the mass spectra with high Al/Fe ratios represent particles containing aluminum originating from the vaporization of spacecraft during atmospheric reentry. Notably, there is no corresponding population with enhanced sodium, which is not used in significant amounts in spacecraft.

More exotic elements such as niobium provide an unequivocal signal of ablation from specific rocket body structures. Fig. 1 shows examples of single-particle mass spectra, one from a typical sulfuric acid particle containing meteoric metals and one from a particle that contains both meteoric metals and elements derived from spacecraft. The signal-to-noise ratio of about 10^4^ in Fig. 1 is routine for mass spectra from the PALMS instrument. Some of the major peaks common to both particles are labeled on the meteoric spectrum (Fig. 2*A*) and peaks that differ due to the presence of metals from spacecraft are labeled in Fig. 1*B*. Over 200 stratospheric particles with clear niobium signals were sampled during the SABRE flights (about 0.1% of the particles). The Nb to Hf ratio derived from the mass spectra of these particles (*SI Appendix*, Fig. S1) matches the C-103 alloy that is used in certain rocket nozzle extensions (11, 12). Particles with niobium or silver almost always contained enhanced aluminum, but there is no such correlation with sodium (Fig. 2).

**Fig. 3.**
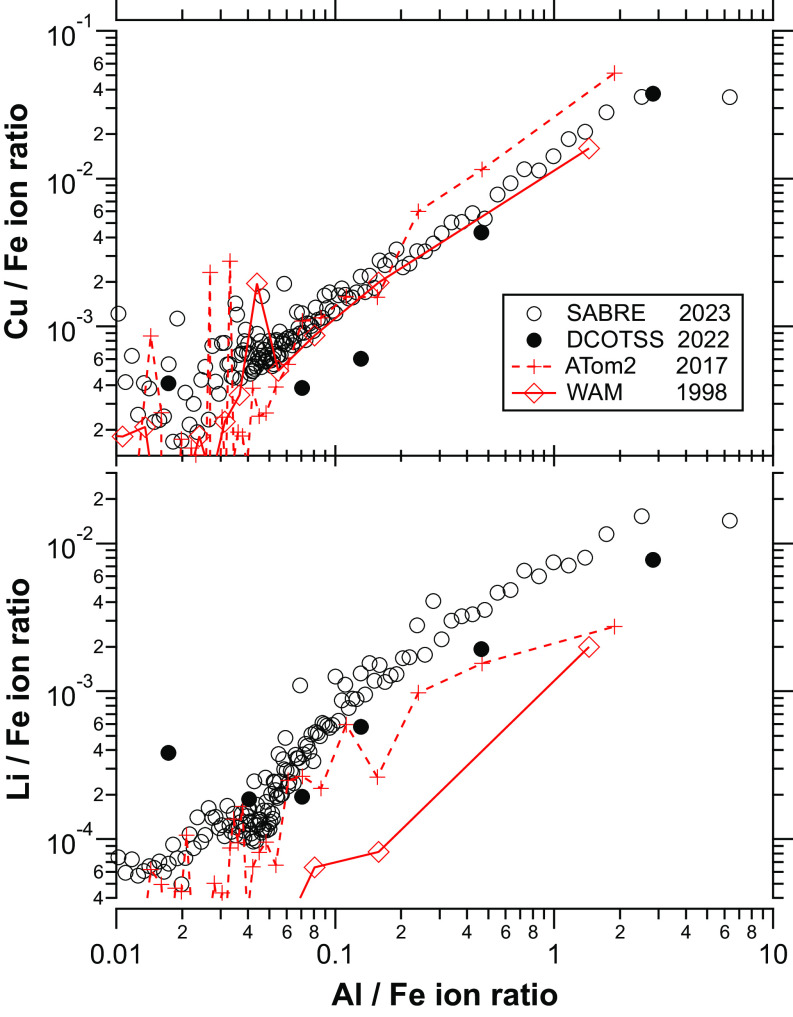
Copper and lithium ion ratios to iron relative to the aluminum to iron ratio. Each point is the average of the ratios from 1,000 (SABRE) or 150 (other missions) mass spectra after sorting the mass spectra by the Al/Fe ion ratio. The scatter in the data at Cu/Fe ratios less than 0.001 is due to small interferences for ^63^Cu such as organic fragment ions.

Quantitation of other metals from spacecraft reentry is provided in Fig. 3, which shows the correlation of copper and lithium with aluminum. There is little copper in meteoroids, but copper is used not only in spacecraft wiring but also comprises about 6% of the widely used AA2219 aluminum alloy (13). By applying laboratory calibrations (*Materials and Methods*) to Fig. 3, we find a copper to aluminum mass ratio of 0.12±0.06 compared to a ratio of about 0.1 in an inventory of reentering material (13). The ratio of copper to aluminum is similar in PALMS data as far back as 1998.

Lithium is also strongly correlated with aluminum (Fig. 3). Applying laboratory calibrations indicates a Li/Al mass ratio in reentry-influenced particles of 0.004 ± 0.002. About 5% of the mass of rocket bodies are now composed of the lightweight AA2195, AA2196, or AA2198 Al-Li alloys that contain up to a few percent lithium (13). Lithium-ion batteries are also increasingly used in spacecraft power systems. Lithium is often identified in spectral emissions from reentering spacecraft as an indication of ablation of structural components (14). There is more lithium per amount of aluminum in the 2023 SABRE and 2022 Dynamics and Chemistry of the Summer Stratosphere (DCOTSS) data than in older data, probably due to increasing use of these Al-Li alloys and lithium batteries.

A detail in the correlations of copper and lithium with aluminum points to a possible small contribution from rocket exhaust. The correlations in Fig. 3 are linear except at the very highest Al/Fe ratios. Examination of such single-particle mass spectra shows that a few are similar to alumina particles emitted by solid propellant rocket motors (15). The vast majority of the mass of alumina particles from solid rocket motor exhaust is contained in a >1 µm mode that quickly sediments from the stratosphere. A lesser submicron mode (16) could remain in the stratosphere with subsequent condensation of sulfuric acid.

Like copper, there is little lead in meteoroids but it was measured in the stratospheric particles with enhanced aluminum. The correlation (*SI Appendix*, Fig. S3) indicates a Pb/Al mass ratio of 0.009±0.005. Because of the high volatility of lead, it is likely that a higher fraction ablates upon reentry than for aluminum.

### The Formation of Particles from Spacecraft Reentry.

Our data provide insights into the processes that produce particles after the vaporization of the various metallic components of spacecraft during reentry. The destruction of satellites, spent rocket stages, and other space debris is a complex process that has been of increasing interest because surviving components present a safety hazard (17). Little attention has been given to the fate of the ablated material (18). Up to 90% of satellites and spent upper stages reentering from orbit are vaporized; lower stages also ablate but to a lesser degree (13, 17).

There are some significant differences between the ablation of meteors and spacecraft. Most of the meteoric mass is deposited at altitudes between 75 and 110 km by a very large number of submillimeter meteoroids (8, 13). Reentering spacecraft, which are larger and moving more slowly, ablate between 40 and 70 km over a ~300 km long footprint (17). There are several hundred major satellite and upper-stage reentry events per year, each one of which deposits up to several tons of mass compared to the microgram quantities from each individual meteoroid.

Almost all of the stratospheric particles with spacecraft metals also contain meteoric metals (Fig. 2*A*). The geographically isolated spacecraft particles (i.e., plumes) mix with the global background of meteoric smoke particles as both are taken up in the atmospheric circulation. We may expect that the metals from reentering spacecraft mix with and condense on meteoric smoke particles that are coming from above, followed by more coagulation during descent into the stratosphere.

That aluminum from spacecraft is generally found in the same particles as niobium and hafnium that is specific to rocket engine nozzles shows that the particles are neither intact bits of melted material nor ablation of the rocket nozzle by hot gases during ascent. The tanks, structures, and rocket engines may ablate at different times during the reentry, yet elements from each are found in the same stratospheric particles. This suggests mixing or coagulation in the cold far-field reentry plume. The mixing during a reentry is not complete, however. For example, the niobium content is highly variable.

The particles containing niobium are almost always distinct from particles containing silver (Fig. 2*A*). Particles with silver often also contained tin and lead (*SI Appendix*, Fig. S2) and had especially high copper content. Niobium and hafnium are markers for the reentry of some rocket nozzles. We hypothesize that silver may be a marker for electronics that are relatively more common in satellites than in spent rocket stages. It appears that diffusion and coagulation are fast enough for some mixing within a given reentry event but not extensive enough to mix the components of different space reentry events into the same particles.

The metals from reentering spacecraft are expected to form oxides upon ablation (17). Whether or not they remain oxides after weeks or months in concentrated sulfuric acid is uncertain. For the most part, PALMS only detects the metal ion and not the molecular form. One exception is that AgCl^+^ was frequently present in spectra containing silver. The F^-^ ion in some negative ion mass spectra (e.g., Fig. 1*B*) may indicate that one of the spacecraft-derived metals forms a fluorine compound.

### Prevalence in the Stratosphere.

**Fig. 4.**
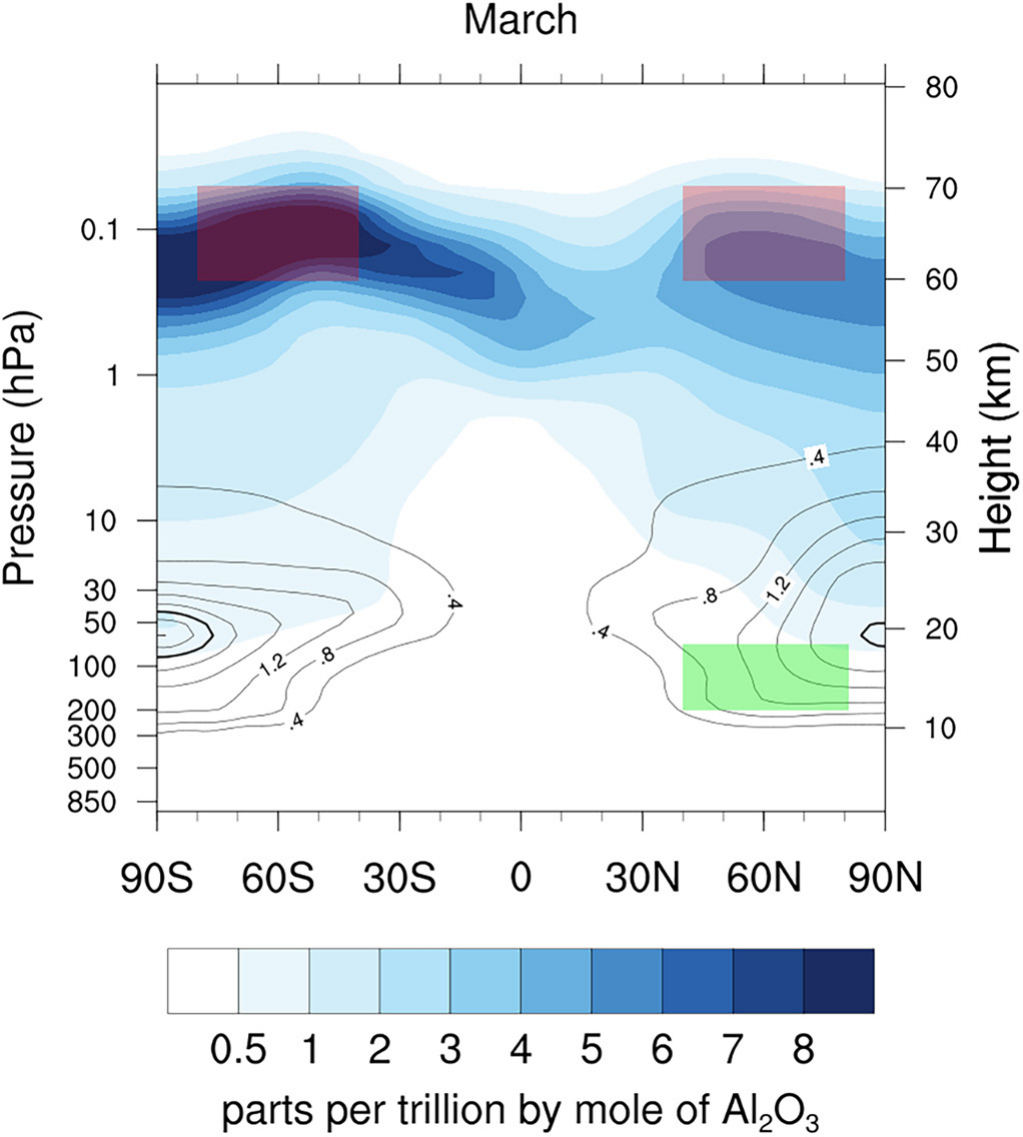
March monthly average concentrations of reentry Al_2_O_3_ for the 15-y reentry emission simulation. Colored filled contours show the mass mixing ratio and the contour lines show the mass density in 10^−16^ g cm^−3^. The bold contour is 2.4 × 10^−16^ g cm^−3^. Red boxes show the assumed ablation region for the simulation, and the green box shows the region in which most of the SABRE sampling occurred. A September average (*SI Appendix*, Fig. S4) also shows descent at high latitudes but with significantly higher concentrations at Southern high latitudes.

The products of both meteoric and spacecraft reentry on a quasi-steady state are concentrated in the polar regions because that is where the global circulation brings descending air from mesosphere into the stratosphere during winter. Fig. 4 shows the March monthly average concentration of a hypothetical reentry aerosol from a multiyear WACCM/CARMA simulation (*Materials and Methods*). Reentry aerosol leaves the emission regions (red boxes) and begins to accumulate between 15 and 30 km poleward of 40° within a matter of months. Meteoric metals follow a similar trajectory: 75% or even more of the sulfuric acid particles in the polar lower stratosphere contain meteoric metals compared to roughly half at lower latitudes.

**Fig. 5.**
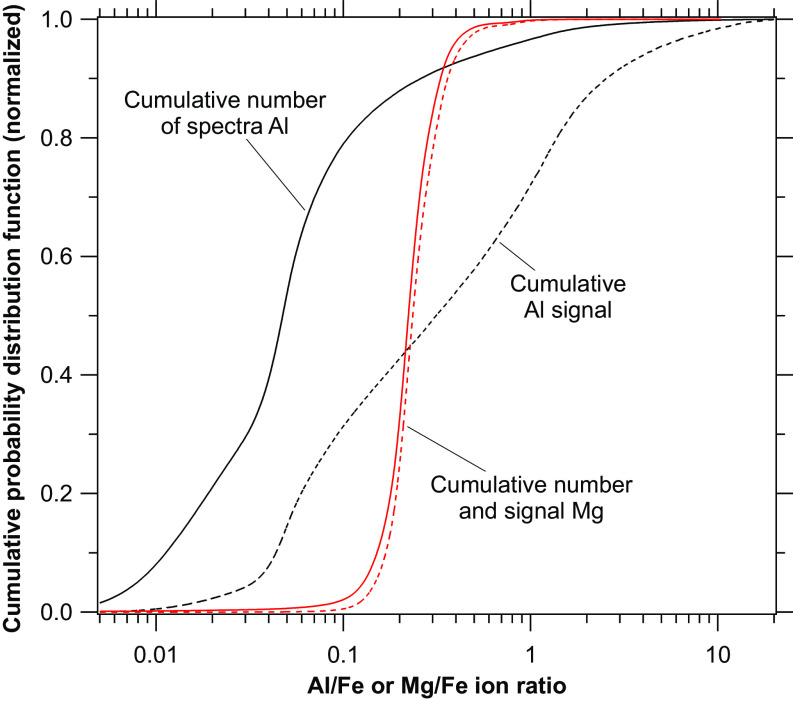
Cumulative probability distributions of the Al and Mg ion signals for particles containing Fe during all SABRE 2023 flights from Alaska (n ≈ 175,000). Particles with an Al/Fe ratio of more than 0.1 have probable spacecraft influence and represent more than 20% of the particles with metals. These Al-enriched particles contain about 70% of the cumulative aluminum signal (dashed). The Mg/Fe distribution is much tighter than the Al/Fe distribution because meteors dominate the source of Mg and Fe.

The 2023 SABRE flights from Fairbanks, Alaska, sampled descending air that was enriched in particles influenced by spacecraft. We can estimate the impact on the broader stratosphere by considering only particles containing meteoric iron. At midlatitudes, roughly half of stratospheric acid particles contain iron from meteors and during SABRE about 20% of such particles contained enhanced aluminum from spacecraft reentry (Fig. 5). Therefore, spacecraft ablation products are present in 10±7% of stratospheric sulfuric acid particles that are larger than about 120 nm diameter. The uncertainty estimate comes from both the threshold for identifying enhanced aluminum and the variability with particle size in the fraction of sulfuric acid particles that contain metals.

The particles are broadly dispersed by the time they have descended into the stratosphere. For example, both niobium and silver were found in widely spaced particles rather than being encountered successively in distinct plumes. The ATom flights in 2017 sampled stratospheric particles in both the Northern and Southern Hemispheres. There were no obvious differences in either the meteoric or spacecraft signatures between the hemispheres, which is broadly consistent with the expected sources.

In our data, the particles with enhanced aluminum from spacecraft contain at least 70% of the total aluminum detected in stratospheric particles (Fig. 5). This is consistent with flux estimates. The flux of aluminum from meteoroids is estimated to be 130 tons y^−1^, of which about 20 tons y^−1^ ablates (8, 13). About 210 tons y^−1^ of aluminum ablates from reentering spacecraft (13). More than 90% of the lithium, copper, and lead are in particles with signatures of spacecraft reentry. These elements are mostly present in the stratosphere only because of the disposal of space debris into the atmosphere.

Elements from the reentry of spacecraft must be detected against the background of meteoric elements and sulfuric acid. Iron is present in significant quantities in spacecraft materials (13) but cannot currently be quantified on top of the larger meteoric contribution. Titanium is also extensively used in spacecraft but is hard to quantify because the abundant ^48^Ti isotope is usually obscured by the SO^+^ fragment of sulfuric acid. In addition to the very clear reentry signals from Li, Al, Cu, Nb, Ag, Hf, and Pb, other elements were detected in occasional mass spectra with especially large peaks from that element. These include Be, Mg, Ti, Cr, Fe, Ni, Zn, Ga, Mo, Cd, In, Sn, Sb, Ba, Ce, Pr, Nd, Ta, W, and Bi.

## Discussion

It is remarkable that the products of spacecraft reentry occurring above 50 km altitude can be measured with such sensitivity in aerosol particles at <19 km altitude. Hafnium is not only detectable but quantifiable relative to niobium. Yet hafnium is a minor alloying element used in only one component (<1% of the mass) of some types of launch vehicles. For novel elements that ionize efficiently, do not have mass spectral interferences, and are dispersed through enough reentry events, a global injection rate of less than 100 kg y^−1^ could be detected. This extraordinary sensitivity is the product of four factors: Sampling only aerosols effectively preconcentrates the metals by a factor of over 10^9^ compared to the gas phase; metals ionize readily; PALMS samples a large number of particles; and the high sensitivity and dynamic range of PALMS allows the measurement of peaks that are less than 0.1% of the total ion signal. The use of any significant quantity of a novel metal in spacecraft construction would soon become measurable in the stratospheric aerosol composition after the reentry and ablation of that material.

We have not identified any definite implications of the presence of these metals in stratospheric sulfuric acid particles, but there are a number of possible effects. One potential effect would be if aluminum and novel elements affect the nucleation of ice or nitric acid trihydrate (NAT). Novel ice nuclei can have a large effect even at low concentrations because polar stratospheric clouds nucleate on a small fraction of the particles (19). Analogues of meteoric inclusions in sulfuric acid have been shown to be ice nuclei (20, 21). Metal cations can also induce efflorescence in aerosol particles (22). The results in this paper prompted us to reanalyze some of our own older mass spectra. We have identified spacecraft reentry particles in ice residuals from high-altitude cirrus sampled in 2002, although not at a notably different frequency than meteoric elements.

There is also a possible impact on the size distribution of the stratospheric aerosol layer. Although the spacecraft reentry metals are mostly found in particles with meteoric material, that does not necessarily mean that the number of particles is constant. Larger particles coagulate less rapidly than smaller particles, so adding anthropogenic material to meteoric smoke can increase the number of particles. If so, the sulfuric acid would be distributed into more numerous but smaller particles with different light scattering and radiative forcing.

With a great variety of metals present, novel stratospheric chemistry or unusual optical properties are possible. The metal concentrations are low enough that there would need to be a catalytic cycle for a significant effect on chlorine partitioning in the stratosphere. Copper is a transition metal for which the spacecraft reentry flux already exceeds the input from meteoroids (13) and will continue to increase. Until the perturbations caused by such aerosols are better understood, they represent a growing uncertainty for the stratospheric aerosol layer.

These mixed meteoritic and spacecraft reentry particles will eventually reach the surface but the mass fluxes are generally small compared to tropospheric sources. For example, the global flux of aerosol lead from spacecraft reentry is less than two tons per year compared to atmospheric emissions of over 700 tons per year from just the United States (23). Copper is one element where spacecraft reentry could be an important source. Reentry elements are concentrated over the poles in the lower stratosphere (Fig. 4) and then deposited to the surface at mid- to high-latitudes. If 10% of the copper vaporized in a future reentry scenario (13) were to be deposited on Antarctica, it could possibly double the concentration of copper in Antarctic snow as roughly estimated from total snowfall (24) and copper in recent snow (25).

At present, the refractory material in stratospheric particles is mostly iron, silicon, and magnesium from the natural meteoric source. However, the amount of material from the reentry of upper-stage rockets and satellites is projected to increase dramatically in the next 10 to 30 y (13, 26, 27). As a result, the amount of aluminum in stratospheric sulfuric acid particles is expected to become comparable to or even exceed the amount of meteoric iron, with unknown consequences for inclusions and ice nucleation.

The space industry has entered an era of rapid growth. With tens of thousands of small satellites planned for low earth orbit (26, 27), that increased mass will be divided into many more reentry events. Given that 10% of stratospheric particles now contain enhanced aluminum, with many more reentry events, it is likely that in the next few decades, the percentage of stratospheric sulfuric acid particles that contain aluminum and other metals from satellite reentry will be comparable to the roughly 50% that now contain meteoric metals.

## Materials and Methods

The PALMS instrument measures the composition of single aerosol particle, including flights on the WB-57, P-3, DC-8, and ER-2 aircraft (28). Data are included here from the WB57 Aerosol Mission (WAM) in 1998, the Atmospheric Tomography Mission (ATom) in 2017, the DCOTSS mission in 2022, and the SABRE flights in 2023. The second deployment of the ATom mission is used in Fig. 3 because it had the most PALMS stratospheric data. A next-generation instrument flown since 2021 uses an sTOF mass analyzer with improved resolution and ion transmission (29) as well as better particle detection optics. WAM and ATom used the older version of PALMS. The updated instruments at Purdue (DCOTSS) and National Oceanic and Atmospheric Administration (SABRE) have almost identical hardware but were operated independently with regard to details such as laser and inlet alignment.

Particles entering a vacuum chamber cross two 405 nm continuous laser beams (one 532 nm laser beam in the 1998 data) that size the particle and provide a trigger for an excimer laser. The lower size limit of particles sampled by PALMS is set by detecting them with scattered light as they fly through the instrument, not the number of ions formed. The updated (older) instrument efficiently measures particles as small as 120 nm (160 nm) geometric diameter and detects a few particles as small as 90 nm (100 nm). There are few particles larger than about 600 nm in the stratosphere when it is unperturbed by volcanic eruptions or other events. A 193-nm laser pulse ablates each particle and creates ions that are analyzed in a time-of-flight mass spectrometer. One distinguishing feature of PALMS compared to other single-particle mass spectrometers is that it obtains mass spectra from over 90% of the particle triggers. This minimizes biases against hard-to-ionize particles.

After acquisition, the signals from high- and low-gain digitizer channels are merged and the baseline restored from the AC coupling of the microchannel plate detector. The 1 Gs s^–1^ sample rate is averaged to 0.33 Gs s^−1^. Peaks are automatically identified and integrated after smoothing to reduce electronic noise. The spectra in Fig. 1 and *SI Appendix*, Fig. S2 have a 35-point binomial smooth applied to the 0.33 Gs s^–1^ data.

The absolute ion signal from a given particle can vary depending on its position in the excimer laser beam and other factors, including the presence of inclusions that may be inconsistently ionized. For identical particles, the ratios between peaks are much more stable than the absolute signals. Therefore, we use ratios of a peak either to another peak or to the total of all peaks in the spectrum. The latter ratio may underestimate the fraction of aluminum from spacecraft reentry in Fig. 5 because a fraction of total ions is not proportional to the added aluminum when the fraction is already high. For example, doubling the aluminum in a particle which is already 50% aluminum raises the percentage to only 67%.

In order to use mass spectra with the most uniform ionization, Figs. 2 and 3 are restricted to particles with diameters less than 500 nm. This includes most stratospheric particles. According to optical particle counter size distributions, less than 2% of ambient particles in the stratosphere during SABRE were larger than 500 nm. A few particles with the largest ^32^S^+^ peaks (>0.3% of the total of all peaks) were also excluded because sulfuric acid is the most difficult atmospherically relevant species to vaporize in a single-particle mass spectrometer (30), and such particles are likely to be incompletely vaporized. In order to analyze small peaks such as the lithium and copper presented in Fig. 3, the total signal from the microchannel plate is required to be at least 10^8^ electrons. Most particles with meteoric or reentry metals have total signals considerably larger than this. For comparison, the spectra in Fig. 1 each have total signals of about 2 × 10^9^ electrons.

The lithium signal in this work is defined only from the ^7^Li^+^ peak because ^6^Li^+^ is often below the detection limit. For lithium and all the elements described here, appropriate corrections are then made for the other isotopes. Aluminum is simply the peak at m/z 27. Iron is defined from ^56^Fe^+^, ^72^(FeO)^+^, and ^54^Fe^+^ after the latter is corrected for ^54^Cr^+^ based on the ^52^Cr^+^ signal. ^63^Cu is defined as the portion of m/z 63 that is larger than m/z 61 after correcting the latter for ^61^Ni and Al(OH)_2_^+^. The corrections are small except when Cu/Fe is less than about 0.001. Only ^63^Cu is used because of the sulfuric acid fragment HSO_2_^+^ at m/z 65. Niobium is defined as the sum of ^93^Nb^+^ and ^109^(NbO)^+^ but only to the extent that m/z 93 is larger than neighboring peaks. The Nb contribution from m/z 109 is limited to the size of the corrected m/z 93. In particular, a large 109 peak without a corresponding 93 peak is considered to have no Nb signal. Silver is defined from the sum of m/z 107 and 109, only when 107 is larger than neighboring peaks and the ratio of 107 to 109 is within about 40% of the isotope ratio. Hafnium is defined as the sum of m/z 177 to 180 when they are larger than neighboring peaks, plus the sum of the oxide peaks at m/z 193 to 196 with the oxide limited to the metal ion peak in a similar fashion to niobium. Metal signals from the few stratospheric spectra with significant ^12^C^+^ peaks are excluded because other organic peaks could confuse the analysis. An analysis of similar peaks in tropospheric particles with frequent organics and dust would need to be much more complex than the formulas here.

We performed PALMS laboratory calibrations by nebulizing various metals in 65 wt% sulfuric acid (balance water), which was used to resemble the bulk composition of stratospheric aerosol. The first standard solution contained 0.5 wt% Fe, 0.5 wt% Mg, 0.05 wt% Al, and 0.05 wt% Na. The second standard solution contained 0.05 wt% Al, 0.1 wt% Cu, and 0.02 wt% Li. Separate solutions were used to facilitate getting all the metals in solution. The calibration for Pb required a different matrix due to the formation of a lead sulfate precipitate in sulfuric acid. Instead, 0.05 wt% Pb and 0.05 wt% Al were dissolved in a 0.019 M ammonium sulfate solution.

The metal-containing standard solutions were nebulized with 0.5 lpm ultra zero grade air, then mixed with an ~19 lpm zero air dilution flow in order to reduce the number concentration and dry the aerosols. The diluted aerosol flow was then passed through an impactor to remove particles > 1 µm prior to their introduction to PALMS.

To obtain the relative sensitivity of the metals, we used ion signal ratios of the same mass peaks for the known solutions as in the analysis of the flight data. Relative sensitivities in ions per unit mass compared to Fe are 0.8 for Na, 1.0 for Mg, and 2.8 for Al. Relative sensitivities in ions per unit mass compared to Al are 1.8 for Li, 0.32 for Cu, and 0.19 for Pb. The statistical uncertainties in the calibrations are <20%, but we estimate a larger uncertainty based on how representative the laboratory particles are of the stratospheric particles. In particular, the calibration particles do not contain silicon or minor metals present in stratospheric particles and their water content differs from the stratospheric particles. Solutions with different combinations of metals than those used for the final calibrations, different particle sizes, and tests with organic contamination changed the relative sensitivities by up to about ±50%. We therefore estimate the uncertainty as a factor of 1.5 to 2.

A multiyear simulation of the transport and stratospheric accumulation of a hypothetical reentry aerosol was performed using the Whole Atmosphere Community Climate Model (WACCM6) (31). The simulation was run for 15 y using repeating year 2000 sea surface temperatures and greenhouse gas emissions. Coupled with WACCM6 is the sectional microphysical model, the Community Aerosol and Radiation Model for Atmospheres (CARMA) (32, 33). Aerosol number and mass in CARMA were tracked in 21 individual size bins starting at 0.01 µm diameter. Estimated present-day satellite reentry emissions of 0.4 Gg y^−1^ of Al_2_O_3_ were used (27) as a surrogate for the density and other properties of all of the reentry material. Ablation was assumed to occur between 60 km and 70 km in the 40 S to 80 S and 40 N to 80 N latitude bands where many satellites now reenter. The ablated material was initialized into the smallest (10 nm) bin. CARMA simulates coagulation and sulfuric acid accumulation onto existing particles.

## Supplementary Material

Appendix 01 (PDF)

## Data Availability

PALMS SABRE data have been deposited in csl.noaa.gov (https://csl.noaa.gov/projects/sabre/data.html; https://csl.noaa.gov/groups/csl7/datasets/data/palms/) (34).
