# Supplementary material for: Metals from spacecraft reentry in stratospheric aerosol particles
Source: Proc Natl Acad Sci U S A. 2023 Oct 16;120(43):e2313374120. doi: 10.1073/pnas.2313374120 (PMC10614211; doi:10.1073/pnas.2313374120)
Supplement: Supplementary file 1 — Appendix 01 (PDF) [file pnas.2313374120.sapp.pdf]

Supplemental figures

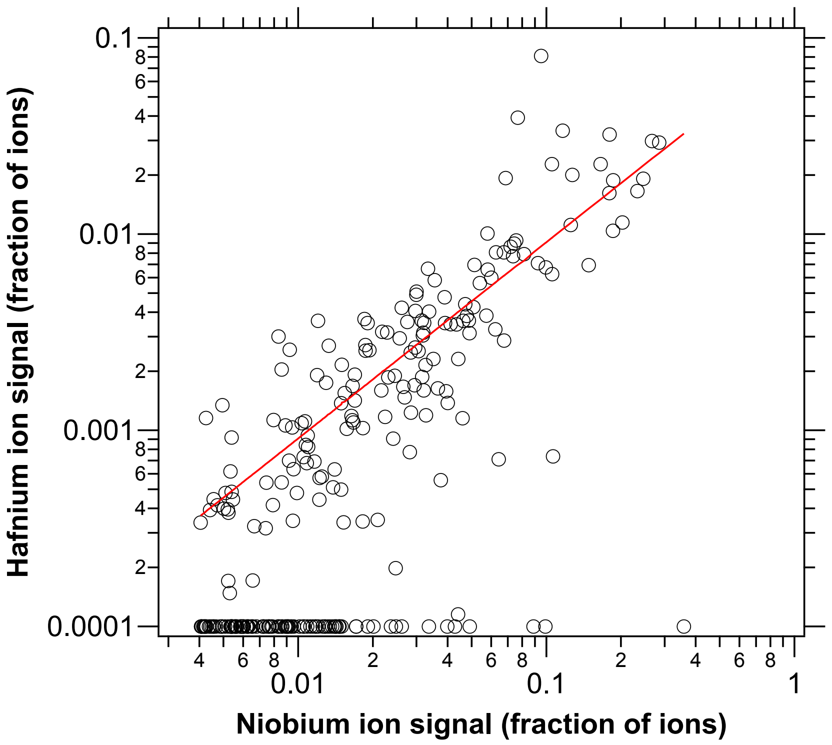

Supplemental Figure 1. Hafnium ion signals as a function of niobium for mass spectra with a niobium signal of at least 0.4% of the total of all peaks. Hafnium below detection limit is plotted at 0.0001. The red line is a linear fit. Based on the PALMS sensitivity to other metals, the sensitivity to hafnium is likely within a factor of 3 of niobium.

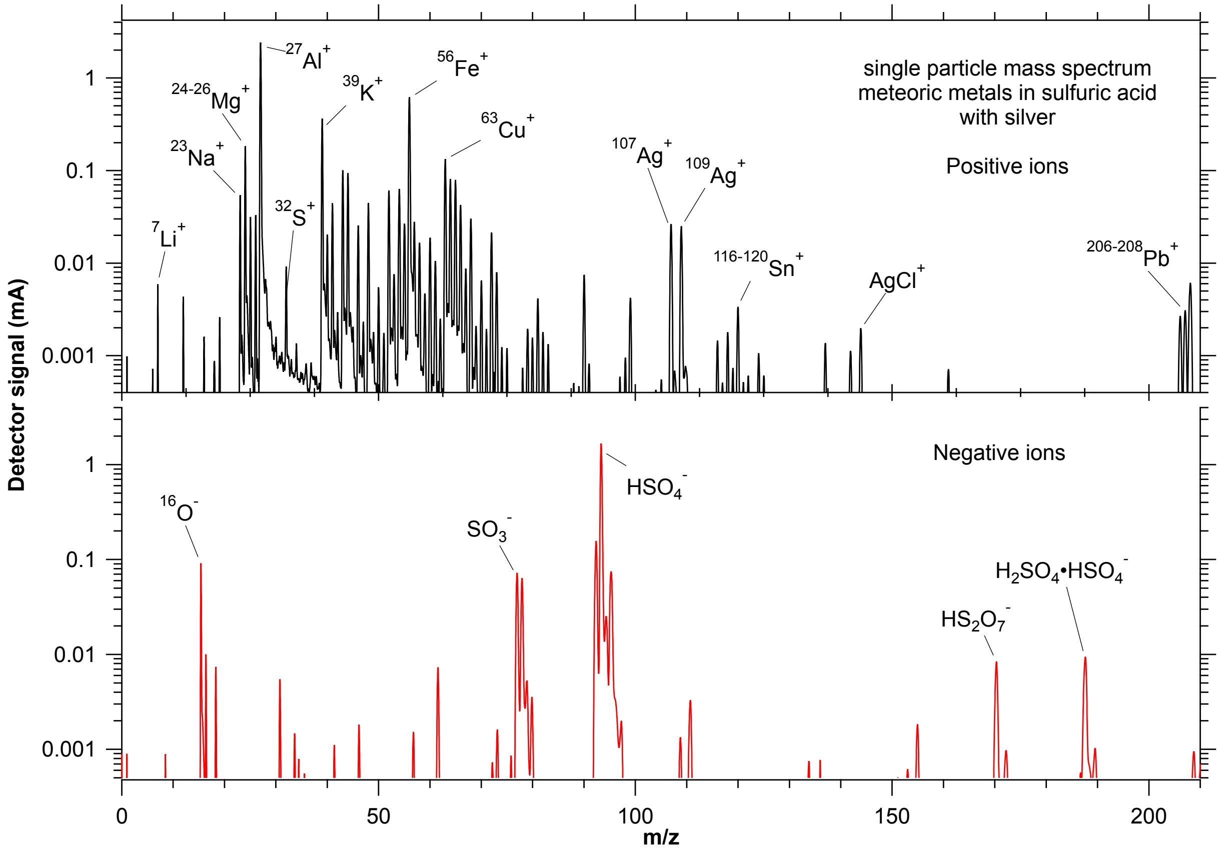

Supplemental Figure 2. An example of a mass spectrum of a stratospheric sulfuric acid particle containing silver along with meteoric metals. The association of silver with lithium, aluminum, copper, tin, and lead was common.

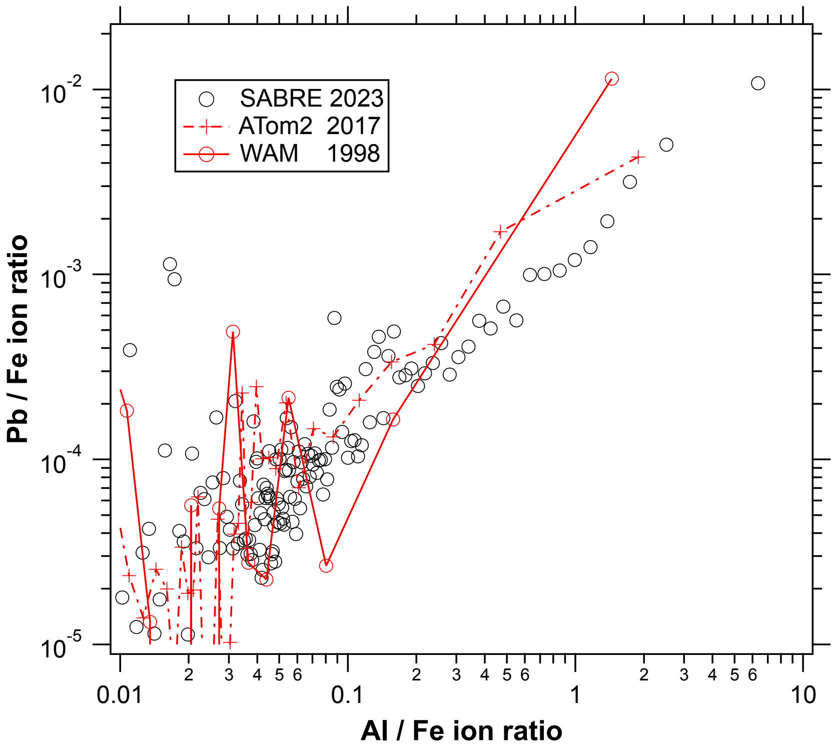

Supplemental Figure 3. Lead signals relative to aluminum in particles which contain iron. Each point is the average of the ratios from 1000 (SABRE) or 150 (other missions) mass spectra after sorting the mass spectra by the Al to Fe ion ratio.

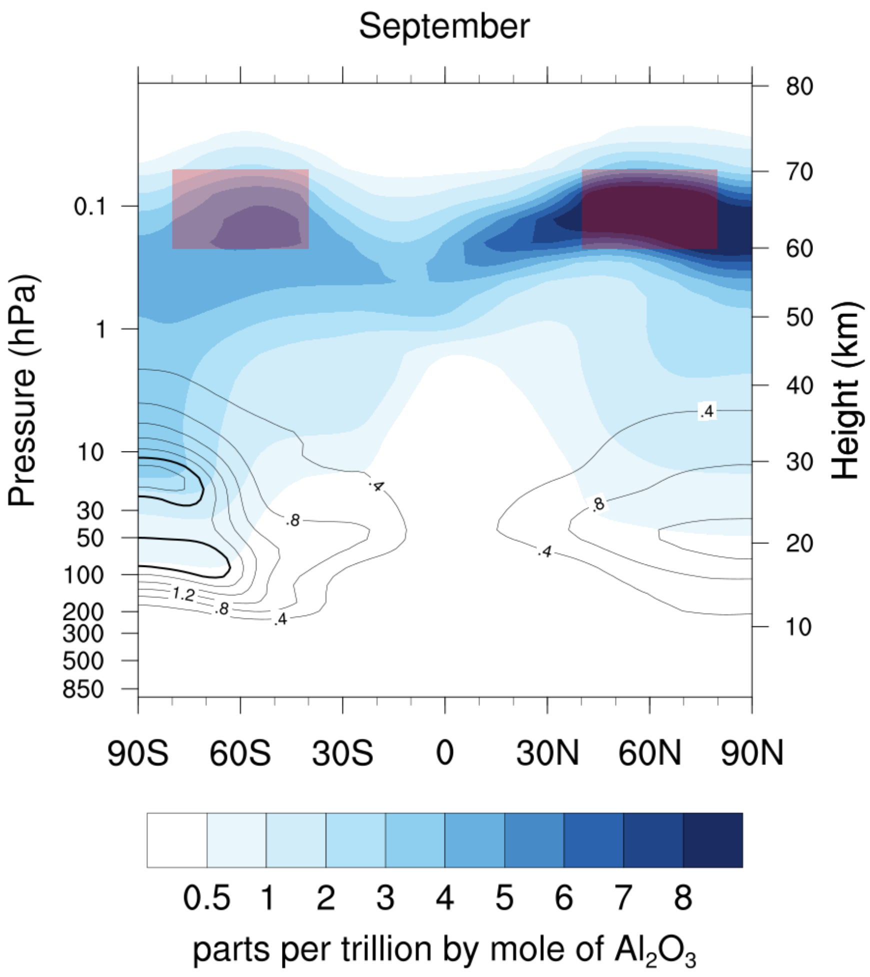

Supplemental Figure 4. The September average concentrations of reentry Al_2O_3 for the 15-year reentry emission simulation. This can be compared to the March average in Fig. 4. Colored filled contours show the mass mixing ratio and the contour lines show the mass density in 10^{-16} g cm^{-3}. The 2.4×10^{-16} g cm^{-3} mass density contour line is shown in bold.
